# Influence of 1-Hydroxyethylidene-1,1-Diphosphonic Acid on the Soft Tissue-Dissolving and Gelatinolytic Effect of Ultrasonically Activated Sodium Hypochlorite in Simulated Endodontic Environments

**DOI:** 10.3390/ma14102531

**Published:** 2021-05-13

**Authors:** Nidambur Vasudev Ballal, Anja Ivica, Pamela Meneses, Raj Kumar Narkedamalli, Thomas Attin, Matthias Zehnder

**Affiliations:** 1 Department of Conservative Dentistry and Endodontics, Manipal Academy of Higher Education, Manipal College of Dental Sciences, Manipal, Karnataka 576104, India; drballal@yahoo.com (N.V.B.); raj231994@gmail.com (R.K.N.); 2 Clinic of Conservative and Preventive Dentistry, University of Zurich, 8032 Zurich, Switzerland; anja.ivica@zzm.uzh.ch (A.I.); pmeneses18@hotmail.com (P.M.); thomas.attin@zzm.uzh.ch (T.A.)

**Keywords:** sodium hypochlorite, root canal, continuous chelation, HEBP, sonochemistry

## Abstract

The addition of Dual Rinse HEDP, an etidronate powder, to a sodium hypochlorite (NaOCl) solution can create a combined single endodontic irrigant with a soft tissue-dissolving and a decalcifying effect, which can replace traditional alternating irrigation with chemically non-compatible solutions. While the short-term compatibility between NaOCl and 1-hydroxyethylidene-1,1-diphosphonic acid (HEDP) has been shown, it remains unclear whether ultrasonic activation of a combined NaOCl & HEDP solution immediately reduces the available chlorine and/or renders the NaOCl ineffective in dissolving organic tissue remnants. This was tested in three experiments: (1) direct activation in test tubes in an ultrasonic bath and then the activation by an ultrasonically oscillating tip (IrriSafe) in (2) an epoxy resin model containing a simulated isthmus filled with gelatin, and (3) extracted teeth with simulated resorption cavities filled with soft tissue. The control solutions were physiological saline and 2.5% NaOCl without HEDP. In (1), available chlorine after 30 s of ultrasonic activation (37 kHz) of test and control solution was assessed, as well as shrimp tissue weight loss in direct exposure. In (2) and (3), the ultrasonic tip was driven at 1/3 of full power using the respective unit, and areas of removed gelatin from the isthmus and tissue weight loss were used as the outcomes, respectively. Experiment (1) revealed no negative impact by HEDP on available chlorine (1), while all three experiments showed a highly significant (*p* > 0.001) synergistic effect, which was not hampered by HEDP, between NaOCl and ultrasonic activation regarding tissue weight loss (1, 3) and dissolution of gelatin (2).

## 1. Introduction

Root canal debridement and disinfection performed in clinics can be performed according to various protocols [1]. The core concept of this task is to clean the root canal system as thoroughly as possible, and then fill it with either a bacteria-tight seal or regenerated tissue. The microanatomy of the root canal system to be treated plays a decisive role in this context. Root canal complexities such as isthmuses and lateral canals can harbor biofilms and soft tissue remnants, which, in turn, can become an avenue of re-infection and source of microbial nutrition in previously treated root canals [2,3]. Consequently, it has become an accepted concept among clinicians that the more complex the endodontic anatomy is, the higher becomes the need for extra measures in the chemo-mechanical root canal debridement. The latter relies heavily on use of a sodium hypochlorite (NaOCl) solution [4]. This is because NaOCl solutions uniquely exert cleansing and anti-biofilm properties [5]. The dissolution of necrotic and infected soft tissue remnants is an important aspect in this context. It is explained by the chlorination and oxidation of proteins by the NaOCl, leading to deproteinization/proteolysis and the concomitant loss of available chlorine in the solution [6]. While in cases with single root canals and simple anatomy, irrigation using through a slim needle that can reach close to working length appears to be enough for a favorable treatment outcome [7], “activation” of the NaOCl solution is recommended in more complex cases and/or to save chair time [8]. Physical methods to improve the penetration of endodontic irrigants and enhance their efficacy include the application of pulsed laser light that is absorbed in the solution or the introduction of a tip into the root canal system oscillating at sonic (up to 6 kHz) or ultrasonic (above 20 kHz) frequency [8]. Activation methods exerting sonic energy are more popular amongst clinicians than laser-activated irrigation [4]. The main reason why NaOCl solutions are ultrasonically activated is to improve their antimicrobial effect and enhance their cleansing power on organic debris and necrotic tissue remnants [9].

Irrigation with a pure NaOCl solution during and after root canal treatment leaves the root canal walls with a smear layer, and inorganic debris can accumulate in canal fins and ramifications [10,11]. Therefore, the additional use of a decalcification agent is recommended. Traditionally, this was performed by alternating between the NaOCl irrigant and a counterpart containing EDTA (ethylenediaminetetraacetic acid) or citric acid [1]. More recently, an etidronate has become available on the dental market, which can be mixed directly into the NaOCl solution to then form an all-in-one irrigant that contains a mild chelator in the form of 1-hydroxyethylidene-1,1-diphosphonic acid or HEDP [12]. This enables clinicians to apply “continuous chelation” in the root canal system during the instrumentation phase and throughout the treatment session. The purported advantage of that is that only one irrigant is necessary for root canal cleaning and disinfection, and irrigation time can be shortened to achieve similar results [13,14]. Moreover, in the continuous chelation approach, the root canal wall is ideally conditioned for adhesive root filling materials, as the canal walls remain free of smear layer, yet the exposed collagen fibrils are still calcified [15]. The disadvantage of continuous combined NaOCl & HEDP irrigation, on the other hand, is that the combined irrigant has to be mixed freshly before treatment and is not stable for storage [12]. Moreover, it has been found that heating the combined NaOCl & HEDP irrigant can speed up the chemical interaction between its components, resulting in quicker loss of available chlorine [12,16]. In addition, because the etidronate is a salt, it increases the density of the combined solution [17], and also its surface tension [13]. This appears not to be a problem in normal usage, as the sodium hypochlorite maintains its main clinical features [18].

Hitherto, one aspect regarding the application of a combined NaOCl & HEDP irrigant for chemo-mechanical root canal treatment has not been investigated in detail. This is its ultrasonic activation, especially with regard to its effect on proteins and soft tissues [6]. It would appear that an ultrasonically activated combined NaOCl & HEDP solution maintains its antibacterial effect [19,20]. However, ultrasonic activation invariably results in an increased temperature of the irrigant in the root canal [21] and may also be hampered by the altered physical features of a combined NaOCl & HEDP solution [14,17]. Moreover, the so-called “sonochemical” effects of NaOCl, i.e., the expediting of chemical reactions in aqueous solution by ultrasound, albeit somewhat enigmatic [22,23], may be linked to increased local temperature in cavitation bubbles [24]. This may accelerate the chemical interaction between NaOCl and HEDP even more, thus leading to a quicker loss in available chlorine [12,16].

In this cascade of experiments, a combined NaOCl & HEDP solution prepared from a commercially available CE-marked etidronate salt (Dual Rinse HEDP, Medcem, Weinfelden, Switzerland) was scrutinized for its usefulness in conjunction with ultrasonic activation. The focus was on the targeted soft tissue-dissolving effect of the NaOCl, which is one of its main clinically desirable features [9,25], and known to be enhanced by ultrasonic activation [26]. Three main experiments were performed:

Firstly, the direct effects of ultrasonic energy on the immediate chemical stability and the soft tissue dissolving effect of the combined NaOCl & HEDP solution were studied in an ultrasonic water bath. Secondly, the ability of this combined solution to clean out gelatin from standardized simulated isthmus areas in epoxy resin models in conjunction with an ultrasonically activated endodontic instrument was tested. Thirdly, the same test was performed in extracted human single-rooted teeth containing simulated resorption cavities filled with soft tissue. The null hypothesis tested was that the addition of the etidronate powder under investigation to a 2.5% NaOCl solution did not alter the desired NaOCl effects in the above three experimental setups, especially in conjunction with ultrasonic activation.

## 2. Materials and Methods

### 2.1. Treatment Groups and Power Analysis

In this cascade of experiments, there were always two times three groups to control for the effect of NaOCl, HEDP, and ultrasonic activation in the different environments. These six groups used in all three experiments were:

Physiological saline solution (0.9% NaCl), no activation vs. ultrasonic activation;
2.5% NaOCl, no activation vs. ultrasonic activation;
2.5% NaOCl containing 9% HEDP, no activation vs. ultrasonic activation.

The sample size for the experiments was calculated based on an effect size of 2, an alpha error probability of 0.05, and 80% power (G*Power 3.1, Heinrich Heine Universität Düseldorf, Germany). Based on this and to be uniform across the experiments, 12 samples per group (*n* = 12) were used in all the tests involving synthetic materials. However, based on the observed treatment effects and the ethical concerns when using an unnecessarily high number of extracted human teeth, the sample size in *Experiment 3* was reduced to *n* = 10 (see Section 2.4).

### 2.2. Ultrasonic Ativation in Water Bath (Experiment 1)

The sodium hypochlorite (NaOCl) solutions were prepared from a more concentrated stock solution with a content of above 4% NaOCl (Scharlab, Sentmenat, Spain). NaOCl solutions were always prepared freshly on the days of the experiment by wt%/wt% dilution in deionized water. The etidronate powder was Dual Rinse HEDP (Medcem). One capsule (0.9 g) of powder was mixed with 10 mL of freshly prepared 2.5% NaOCl per application.

The test and control solutions in this and all the following experiments were used at ambient temperature (23 °C). Their available chlorine was titrated using a 0.1 mol/L sodium thiosulfate solution (Huberlab, Aesch, Switzerland) in a titration apparatus (Metrohm 665 Dosimat Titrator, Zofingen, Switzerland). Solution temperatures were assessed using a calibrated pocket thermometer with a slim steel probe (Checktemp 1, Hanna Instruments, Woonsocket, RI, USA). In this experiment, 1 mL of test or control solution was kept in a 2 mL safelock microcentrifugation tube (Epperdorf; Hamburg, Germany). Tubes were suspended in an ultrasonic water bath (SW 3H, Sono Swiss, Ramsen, Switzerland) containing tap water at 38 °C. This water bath had an integrated water temperature control and an ultrasonic frequency of 37 kHz. The tubes were kept in vertical position in a rack (Nalgene Microcentrifuge Tube Rack, Fisher Scientific, Waltham, MA, USA) and activated or not (depending on group assignment) for 30 s.

To assess the effect of ultrasonic energy on the tissue dissolution efficacy of the solutions under investigation, pre-cooked cocktail shrimp meat (Costa, Emden, Germany) was used. Shrimp meat was cut to a standardized size, blotted dry, and pre-weighed in a precision balance (AT 261, Mettler Toledo, Greifensee, Switzerland). Weighing of the tissue pieces here and in *Experiment 3* was done under environmental conditions (23 °C and 40% relative humidity). Subsequently, tissue pieces were individually suspended in 1 mL of test or control solutions. The microcentrifugation tube containing the soft tissue was then suspended in the water bath as described above, and activated or not for 30 s. The overall exposure time of the shrimp tissue to test or control solutions (including positioning in the water bath and removing from it) was 1 min. Subsequently, the soft tissue pieces were washed in a 0.1 mol/L sodium thiosulfate solution (Huberlab, Genève, Switzerland) and NaCl 0.9% (B. Braun Melsungen AG, Melsungen, Germany) blotted dry, and weighed again. The weight loss of the tissue samples after exposure to the test/control solutions and treatments was used as the outcome variable.

### 2.3. Cleaning of Isthmus Areas in Epoxy Resin Models (Experiment 2)

**Figure 1:**
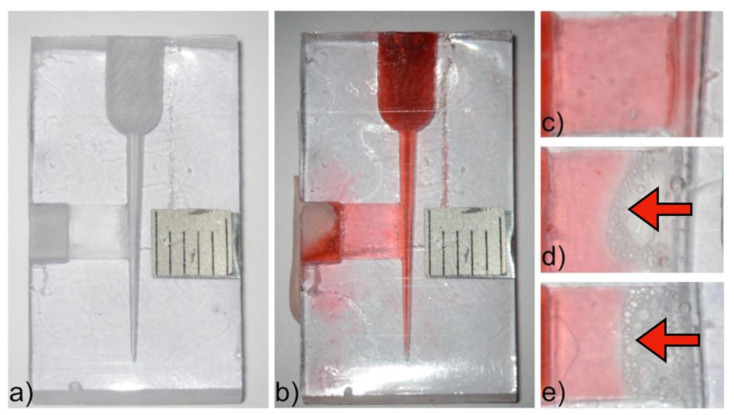
(**a**) A finger spreader and a matrix band were used to create models representing a root canal with a lateral isthmus. (**b**) The model was filled with gelatin mixed with red food dye. (**c**) Irrigation with physiological saline was used as a negative control. (**d**) When NaOCl was used as an irrigant and activated with am ultrasonically oscillating tip for 30 s, 43.2 ± 8.7% of isthmus surface was cleaned. (**e**) Adding HEDP to NaOCl did not affect cleaning efficacy (47.9 ± 9.4% cleaned, *p* > 0.05), as can be appreciated by comparing panels (**d**) and (**e**) (arrows).

Transparent models were prepared using a two-component epoxy resin that cures at room temperature (Loctite Stycast 1266, Henkel, Düsseldorf, Germany). This model was an alteration of a previously published model to assess ultrasonic irrigation [27], with the incorporation of a simulated isthmus filled with a hydrogel in the form of colored gelatin as a soft tissue surrogate [28]. In brief, a D-size finger spreader of 25 mm length (Dentsply Mailefer, Ballaigues, Switzerland) was inserted to a penetration depth of 22 mm in a wax mold (15 × 26 × 8 mm). For an isthmus, a folded 0.05 mm thick matrix band (Ivory, Omnident, Rodgau, Germany) was connected to the spreader using wax at 7 mm from the spreader tip. This created a simulated isthmus of 0.1 mm depth and an area of 4 mm height and 5 mm width (Figure 1). A millimeter scale was glued to the opposite side of the wax frame. Resin and hardener were mixed in a clean container in the weight ratio 3.57:1. The mixture was vacuumed to remove entrapped air and then poured into a wax mold. The models were left overnight at room temperature to ensure setting. An access cavity was then created by drilling 6 mm into the simulated main canal. The remaining main canal in these models thus had a length of 16 mm, apical diameter of 0.35 mm and a taper of 6% (Figure 1a). The canal and the isthmus were filled with a hydrogel prepared as a 20% gelatin solution (Merck, Darmstadt, Germany) dissolved in deionized water with addition of a 1% red food dye (2 mL). A syringe with hydrogel was kept in a warm water prior to delivery into the models. To close the isthmus opening soft putty (Delta-SP 80, Intertrading Dental AG, Rotkreuz, Switzerland) was used. The models filled with hydrogel were randomly assigned to one of the six groups (see Section 2.1). Each model was used twice per group.

In the ultrasonically activated solutions, 2.5 mL of irrigant was administered for 30 s, followed by passive ultrasonic activation for 30 s [29]. The ultrasonic tip (IrriSafe, Satelec, Acteon Group Ltd., Norwich, UK) was driven by a piezoelectric ultrasonic device (Satelec) set at 1/3 of full power. The ultrasonic tip was placed 1–2 mm short of working length and activated passively within the root canal. In the groups without ultrasonic activation, 2.5 mL of irrigant was administered during 30 s without ultrasonic activation. All the irrigating solutions were introduced into the canal using a 30-gauge side vented stainless-steel needle (Vista Apex Dental Products, Racine, WI, USA). The needle tip was inserted to 1 mm short of full simulated root canal length. 

Images were obtained using dental microscope OPMI PROergo (Zeiss, Jena, Germany) equipped with a digital camera (Nikon D800, Tokyo, Japan). The removal of gelatin from an isthmus was calculated using ImageJ (National Institutes of Health, Bethesda, MD, USA). First, the whole isthmus area was calculated and then the threshold for color was adjusted so that it reflected the remaining gelatin. The percent area cleaned of the gelatin was calculated using the “Analyze Particles” tool. 

After irrigation and imaging, the gelatin was cleaned from the models using hot water and a syringe. Subsequently, the clean models were filled again with hydrogel, randomized, and used for another cycle of irrigation. Each irrigation condition was repeated 11 times (*n* = 12).

### 2.4. Soft Tissue Dissolution from Simulated Resorption Cavities in Human Root Canals (Experiment 3)

Ethical clearance for the use of human extracted teeth for the experiments performed in the present study was obtained from the institutional ethics committee (Kasturba Medical College and Kasturba Hospital, 256/2021). The internal resorption cavity model was adopted from a published paper on root filling [30]. All these teeth were extracted for reasons not related to this study. Sixty extracted human single rooted teeth with fully formed apices containing one straight single root canal were selected. All the teeth were radiographed and analyzed under magnifying loupes (EyeMag Smart, Carl Zeiss, Oberkochen, Germany) to verify the presence of a single canal with fully formed apex, and the absence of intra radicular resorption, caries, cracks or root canal filling. Superficial soft tissues were removed with a curette and the teeth were stored in 0.2% sodium azide (Millipore Sigma, St. Louis, MO, USA) at 4 °C until use. The teeth were then decoronated to standardize the root length. Working length was established by inserting a size 10 K file (Dentsply Sirona Endodontics, Ballaigues, Switzerland) into each root canal until it was just visible at the apical foramen (observed using magnifying loupes) and by subtracting 1 mm from the recorded length. Chemo-mechanical preparation was performed using ProTaper^®^ universal nickel titanium rotary instruments (Dentsply Sirona), and the canals were instrumented to size F3. Irrigation was performed using 5 mL of a 2.5% NaOCl solution for 1 min between each instrument change. Final rinse was performed with 5 mL of 17% EDTA (Vista Apex Dental Products) for 1 min to remove smear layer. The canals were then dried with sterile paper points (Dentsply Sirona). Longitudinal reference grooves of 0.5 mm depth were first made on the buccal and lingual surfaces of middle third of the root to serve as a guide, indicating the standard locations where simulated resorption cavities were to be made in the root canal. Each specimen was then embedded in Eppendorf tubes (Merck, Darmstadt, Germany) using a putty impression material (3M ESPE, St. Paul, MN, USA). Once the putty impression material was set, the roots were removed from the vials to enable assembly and reassembly of the specimens. Each root was then sectioned longitudinally into two halves using a diamond disc (Horico Dental, Berlin, Germany) under water cooling (Supplementary Materials Figure). Semicircular cavities were then made in the root canal wall in the middle third of each root half using no. 6 round diamond point (Horico Dental) operated at slow speed under water cooling. The bur was marked at 2 mm from its tip using a permanent marker, in order to ensure precise depth of the cavity. The depth of simulated resorption cavity in the re-assembled roots was thus standardized to 4 mm. The cavities were irrigated with 5 mL of 2.5% NaOCl for 1 min followed by 5 mL of 17% EDTA (Vista Apex Dental Products) for 1 min to remove the smear layer formed. Finally, cavities were flushed with 5 mL of deionized water for 1 min and dried using paper points (Dentsply Sirona).

Freshly extracted shrimp meat was employed for the experiment to simulate pulp tissue. The tissue samples used were standardized in weight and placed in the simulated resorption cavities of both the halves of each root. Before weighing, each piece of the tissue was blotted on a Whatman filter paper (Merck), dried, and then weighed using a precision balance in an airtight container (AT 261, Mettler Toledo, Genève, Switzerland). Both the root halves were then reassembled using a light curing resin barrier (OpalDam, Ultradent Products, South Jordan, UT, USA). Care was taken to maintain patency by placing a F3 protaper gutta percha point (Dentsply Sirona) between canal the two root sections. The apex of each root was closed with sticky wax to simulate a closed-end system. A plastic tube of 3 mm height was glued at the cementoenamel junction of each root which acted as a reservoir for irrigating solution. Each root was then be placed in their respective Eppendorf vials, and the samples were randomly divided into six groups (*n* = 10) based on irrigation regimen as described above (see Section 2.3).

After the irrigation protocols, each root canal was irrigated with 5 mL of saline to remove any precipitate that might have formed. The roots were then removed from the Eppendorf vials and separated to remove and weigh the soft tissue contained in the simulated resorption cavities. Prior to weighing, all the soft tissue specimens were blotted on a Whatmann filter paper (Merck). All the samples were weighed by a single investigator who was unaware of the test solutions used.

### 2.5. Statistical Analysis

All data presented here were normally distributed (Shapiro–Wilk test). Chemical assessments were performed in triplicates and data are presented as means and standard deviations. Based on the error of measurement, the data is presented to one digit. For all other experiments the influence of the two independent variables “type of irrigant” and “ultrasonic activation” were computed using two-way analysis of variance (ANOVA). Subsequently, mean values were compared between all six groups by one-way ANOVA, followed by Tukey’s highly significant difference (HSD) test. The alpha-type error was set to 5%.

## 3. Results

### 3.1. Ultrasonic Activation in Water Bath (Experiment 1)

When solutions (23 °C) were placed in microcentrifuge tube into an ultrasonic bath (38 °C) and activated (test) or left non-activated for 30 s each, there was no difference in the available chlorine that was assessed immediately thereafter, indicating that the ultrasonic acitvation of the irrigant per se did not trigger a chemical reaction between the HEDP and the NaOCl (Table 1) under these conditions.

Subsequently, the effect of test and control solutions on the dissolution of shrimp meat as a simulated pulp tissue was tested in this water bath. The shrimp tissue pieces were fairly uniform, with an average weight of 113 ± 5 mg. In terms of their weight loss when subjected to the treatments under investigation, there was a highly significant effect of ultrasonic activation and type of irrigant (two-way ANOVA, *p* > 0.001, each). Inter-individual comparisons between groups revealed that the ultrasonic treatment with NaOCl in the solution caused the statistically highest weight loss, while the presence of HEDP in the solution had no impact (Table 2).

### 3.2. Cleaning of Isthmus Areas in Epoxy Resin Models (Experiment 2)

**Figure 2:**
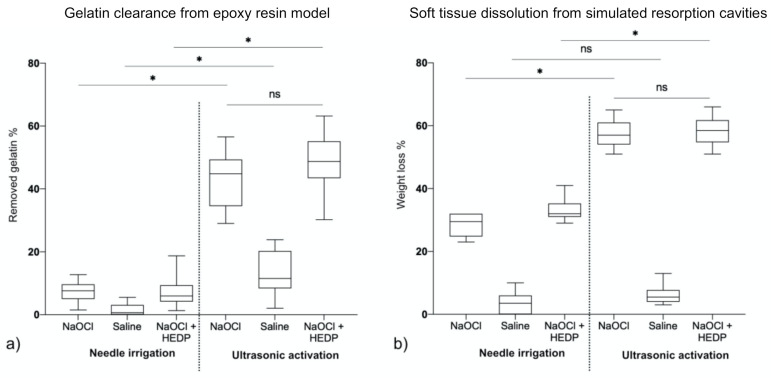
Box plots with error bars indicating maximum and minimum values. (**a**) *Experiment 2:* Percentage of removed gelatin after different irrigation protocols (*n* = 12). Ultrasound activation significantly (*p* > 0.001) improved cleaning of the simulated isthmus in the epoxy resin model. Adding HEDP to NaOCl did not change the efficacy of cleaning (*p* > 0.05). (**b**) *Experiment 3:* Similar results were obtained with the soft tissue dissolution from simulated resorption cavities in human root canals, with the only difference that mere irrigation with solutions containing NaOCl did dissolve some tissue. Asterisks denote significant differences between data sets at the 5% level, ns = non-significant, *p* > 0.05.

To assess the cleaning efficacy of different irrigation protocols, epoxy resin models (Figure 1a) were filled with gelatin (Figure 1b). A highly significant effect of ultrasonic activation and type of irrigant on gelatin removed from the isthmus was observed (two-way ANOVA, *p* > 0.001, each). Needle irrigation with saline was used as a negative control (Figure 1c). With needle irrigation using NaOCl, no major removal of gelatin was detected, which was in contrast to the effect of NaOCl and ultrasound (Figure 1d) (*p* > 0.001). Similar results were observed when HEDP (Figure 1e) was used as an irrigant, with no negative impact of HEDP compared to the pure 2.5% NaOCl solution. (*p* > 0.05, Figure 2a).

### 3.3. Soft Tissue Dissolution from Simulated Resorption Cavities in Human Root Canals (Experiment 3)

The results in this experiment were comparable to those on soft tissue dissolution in the ultrasonic bath and also the removal of the gelatin from the simulated isthmus areas in *Experiment 2* (Figure 2). Again, both ultrasonic activation and type of irrigant had a highly significant effect (two-way ANOVA, *p* > 0.001, each). However, mere irrigation with NaOCl and NaOCl & HEDP did dissolve some tissue (Figure 2b), while it had almost no effect on the removal of gelatin from the simulated isthmus (Figure 1a). Again, the addition of the etidronate did not impact the effectiveness of the NaOCl solution, neither when used passively nor when activated using the ultrasonic tip (*p* > 0.05).

## 4. Discussion

The current study showed in three independent experiments that the combination of a NaOCl solution with HEDP does not interfere with the possibility to activate the NaOCl ultrasonically. Moreover, this study confirmed the synergistic effect between NaOCl and ultrasound.

This investigation is limited by the fact that it was a laboratory study with outcomes that may or may not be related to clinical treatment. Nevertheless, an attempt was made to use different modes of ultrasonic activation as well as different organic materials to investigate the dissolving NaOCl effects under ultrasonic activation. The first experiment was on the direct activation of irrigants in an ultrasonic water bath, where cavitation is the main driver. Sonochemical effects are related to the ultrasonic power used, the presence of bubbled gas, temperature, solvent composition, and reaction volume [31]. These effects have been described for ultrasonic baths as used here, which are normally operated at 20–40 Hz. However, in the confined area of a root canal system, when irrigants are activated by an ultrasonically activated tip or file, multiple effects come into play, and the results must be interpreted as a combined result of cavitation and acoustic streaming [32]. The use of an ultrasonic bath allowed the exact measurement of the available chlorine because of higher irrigant volumes compared to the few µL available in a root canal, which make iodometric titration impossible [33]. Subsequently, soft tissue pieces were added to this system, to see whether the NaOCl would perhaps lose its ability to dissolve them in the presence of HEDP. This could not only be due to a loss in available chlorine, but also because of the altered physical properties of the combined NaOCl & HEDP solution compared to the pure NaOCl counterpart [13,17]. Shrimp meat was used as the surrogate for pulp tissue here and in the third experiment, because it has a similar texture, which may be due to its high proteoglycan content [34,35], and is easily available.

The two further experiments (*Experiments 2* and *3*) used epoxy resin models and extracted human teeth, respectively. This was to test an ultrasonic tip specifically designed for passive ultrasonic activation [36] in a simulated clinical environment. In clinical dentistry, various tools are now available to sonically or ultrasonically activate the irrigants in root canals. However, PUI [29], later also termed UAI for ultrasonically activated irrigation, remains to be the gold standard [4,9,37]. Its mode of action is still not fully elucidated and appears to be influenced by varios factors, such as the power, the type of file that is activated, and the available space in the root canal [32]. Cavitation appears not to be the main effect in this context but may still occur [32]. Cavitation could technically speed up the chemical interaction between HEDP and NaOCl. As was shown here, however, that appears not to occur under current conditions. *Experiments 2* and *3* represented the standard clinical setting in terms of ultrasonic power recommended by the manufacturer.

The observed synergistic effect of NaOCl and ultrasound is in conjunction with published data by other authors, who used Rhodamin B as an indicator [23], or a similar epoxy resin model as we did in *Experiment 2* [26]. The slight difference in outcome with these experiments was that mere irrigation with irrigants containing NaOCl did dissolve some shrimp tissue in the natural teeth, while they had little effect in removing the gelatin from the simulated isthmus (Figure 2). The explanation for this is probably the difference in contact surface. It would appear that the cleaning of a lateral isthmus is almost a pure function of ultrasonic effects [26], while the tissue pieces in the simulated resorption cavities were more exposed to the non-activated irrigant that was contained in the root canal. This would give further support to use epoxy resin models containing simulated canal irregularities filled with a hydrogel as a soft tissue surrogate to study endodontic irrigant activations schemes. Future studies could use such models to assess whether other irrigating schemes are similarly efficient as the PUI setup used here, and how safe they are in terms of over-irrigation in an open system with a simulated apical lesion.

## 5. Conclusions

Under the conditions of these experiments, the synergistic effect between ultrasonic activation of the irrigant and the NaOCl that was contained therein was confirmed, resulting in an enhanced effect on soft tissue and gelatin. The addition of HEDP from an etidronate salt did not hamper this effect. Consequently, none of the current findings speak against using a combined NaOCl & HEDP solution for ultrasonically activated root canal irrigation.

## Figures and Tables

**Table 1:** Amount of available chlorine as determined by iodometric titration when room-temperature (23 °C) irrigants were placed in the ultrasonic water bath (38 °C, 37 kHz) and were then activated or left non-activated for 30 s.

Irrigant	Ultrasonic Activation (30 s)	Temperature (°C) ^1^	NaOCl wt% ^1^
2.5% NaOCl & water ^2^	No	28.5 ± 0.1	2.3 ± 0.0
2.5% NaOCl & 9% HEDP	No	28.7 ± 0.3	2.3 ± 0.0
2.5% NaOCl & 9% HEDP	Yes	30.1 ± 0.2	2.3 ± 0.0

^1^ Mean values of triplicates ± standard deviations; ^2^ to compensate for HEDP weight.

**Table 2:** Soft tissue weight loss when shrimp tissue pieces were suspended in test or control irrigants in Eppendorf tubes placed in the ultrasonic water bath (38 °C, 37 kHz) and were then activated or left non-activated for 30 s.

Irrigant	Ultrasonic Activation (30 s)	Weight Loss (%) ^1^	Statistics ^1^
Saline	No	0.6 ± 0.2	A
	Yes	3.1 ± 0.2	A
2.5% NaOCl	No	8.5 ± 0.3	B
	Yes	19.6 ± 5.7	C
2.5% NaOCl & 9% HEDP	No	9.9 ± 1.1	B
	Yes	17.8 ± 6.9	C

^1^ Connecting letters report: data groups not connected by the same letter are significantly different (ANOVA, Tukey’s HSD, *p* > 0.05).

## Data Availability

Data are available upon request by the authors.

## Supplementary Materials

The following are available online at https://www.mdpi.com/article/10.3390/ma14102531/s1. Figure S1: (**a**) Depiction of a longitudinally sectioned root. Semicircular cavities were pre-pared in the root canal wall in the middle third of each root half using no. 6 round dia-mond operated at slow speed under water cooling. (**b**) After placing the pre-weighed soft tissue pieces into the simulated internal resorption cavities, the root halves were reassem-bled using a light curing resin barrier. Care was exercised to maintain canal patency by placing a F3 ProTaper gutta percha point between the two root sections. The apex of each root was closed with sticky wax to simulate a closed-end system.
