# Supplementary material for: Influence of 1-Hydroxyethylidene-1,1-Diphosphonic Acid on the Soft Tissue-Dissolving and Gelatinolytic Effect of Ultrasonically Activated Sodium Hypochlorite in Simulated Endodontic Environments
Source: Materials (Basel). 2021 May 13;14(10):2531. doi: 10.3390/ma14102531 (PMC8152752; doi:10.3390/ma14102531)
Supplement: Supplementary file 1 [file materials-14-02531-s001.zip › materials-1202743-supplementary.pdf]

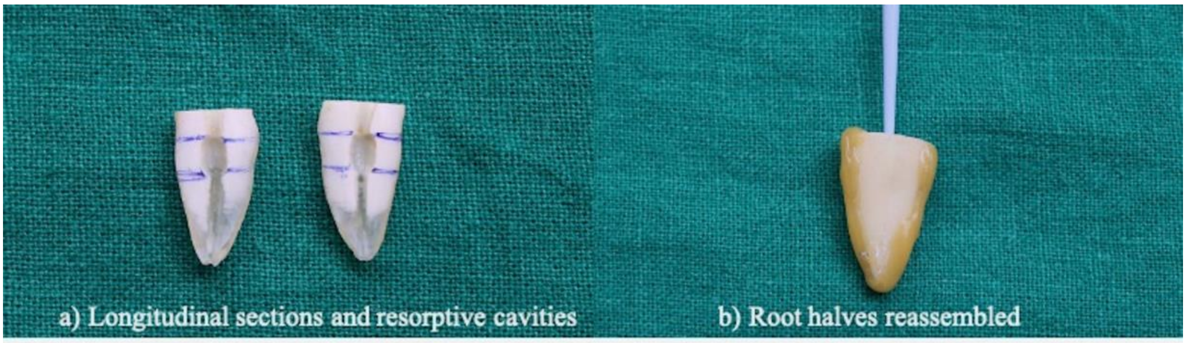

Figure S1. **(a)** Depiction of a longitudinally sectioned root. Semicircular cavities were prepared in the root canal wall in the middle third of each root half using no. 6 round diamond operated at slow speed under water cooling. **(b)** After placing the pre-weighed soft tissue pieces into the simulated internal resorption cavities, the root halves were reassembled using a light curing resin barrier. Care was exercised to maintain canal patency by placing a F3 ProTaper gutta-percha point between the two root sections. The apex of each root was closed with sticky wax to simulate a closed-end system.
